# Investigation of Nitridation on the Band Alignment at MoS_2_/HfO_2_ Interfaces

**DOI:** 10.1186/s11671-019-3020-0

**Published:** 2019-05-29

**Authors:** Ya-Wei Huan, Wen-Jun Liu, Xiao-Bing Tang, Xiao-Yong Xue, Xiao-Lei Wang, Qing-Qing Sun, Shi-Jin Ding

**Affiliations:** 1 State Key Laboratory of ASIC and System, School of Microelectronics, Fudan University, Shanghai, 200433 China; 2 Key Laboratory of Microelectronics Devices & Integrated Technology, Institute of Microelectronics, Chinese Academy of Sciences, Beijing, 100029 China

**Keywords:** Nitridation treatment, Band alignment, Few-layer MoS_2_

## Abstract

The effect of nitridation treatment on the band alignment between few-layer MoS_2_ and HfO_2_ has been investigated by X-ray photoelectron spectroscopy. The valence (conduction) band offsets of MoS_2_/HfO_2_ with and without nitridation treatment were determined to be 2.09 ± 0.1 (2.41 ± 0.1) and 2.34 ± 0.1 (2.16 ± 0.1) eV, respectively. The tunable band alignment could be attributed to the Mo-N bonding formation and surface band bending for HfO_2_ triggered by nitridation. This study on the energy band engineering of MoS_2_/HfO_2_ heterojunctions may also be extended to other high-k dielectrics for integrating with two-dimensional materials to design and optimize their electronic devices.

## Background

Currently, layered transition metal dichalcogenides (TMDCs) have aroused great interest due to their fascinating properties for potential applications in modern electronics and optoelectronics [1, 2]. In particular, molybdenum disulfide (MoS_2_) has been attracting considerable attention as a promising channel material for continuing the scaling beyond the 7-nm technology node [3, 4]. Structurally, the MoS_2_ crystal is built up of one hexagonally arranged Mo plane, sandwiched by two hexagonally arranged S planes. A triangular prismatic arrangement was formed via the covalently bonded S-Mo-S units [5, 6]. MoS_2_ possesses a layer-dependent bandgap, varying from a direct bandgap (1.8 eV) for single-layer (SL) MoS_2_ to an indirect bandgap (1.2 eV) for bulk MoS_2_ [7]. Dissimilar to graphene with a zero bandgap, the thickness-dependent modulation of bandgaps motivated the exploration of MoS_2_ in optical and electrical devices [3, 8]. Based on the physics of MoS_2_, the density of states of few-layer MoS_2_ is triple that of single-layer MoS_2_, resulting in high drive currents in the ballistic limit [8]. In this context, few-layer MoS_2_ may deliver significant advantages for transistor applications than SL MoS_2_ [3].

On the other hand, the electronic devices based on traditional silicon dioxide dielectrics are approaching the physical limit because of its low dielectric constant [9]. To obtain a thin equivalent oxide thickness (EOT), it is crucially important to integrate high-k dielectrics with MoS_2_. To date, many high-k dielectrics have been investigated with MoS_2_, including Al_2_O_3_, ZrO_2_, HfO_2_, and h-BN [10–14]. DiStefano et al. obtained the respective conduction and valence band offsets of 3.3 ± 0.2 and 1.4 ± 0.2 eV for few-layer MoS_2_ grown by oxide vapor deposition on amorphous BN [13]. Tao et al. reported that the conduction band offset (CBO) for the monolayer MoS_2_/Al_2_O_3_ (ZrO_2_) heterojunction was deduced to be 3.56 eV (1.22 eV), while the valence band offset (VBO) was 3.31 eV (2.76 eV) [15]. And a CBO of 2.09 ± 0.35 eV and VBO of 2.67 ± 0.11 eV at the MoS_2_/HfO_2_ interface were reported by McDonnell et al. [12]. Among these gate dielectrics, HfO_2_ was considered to be one of the most promising candidates owing to its high dielectric constant (k ∼ 20), compatibility with poly-SiGe, TaN gates, and polycrystalline silicon gate [16]. However, HfO_2_ has a poor thermal stability, large leakage current, high oxide trap density, interface trap density, etc. [17]. These limitations have motivated extensive investigations of searching passivation techniques, such as interface nitridation or fluorination treatment technologies [18, 19]. In this work, we studied the energy band alignments of few-layer MoS_2_ on HfO_2_ dielectrics with and without plasma nitridation, in which the effect of surface nitridation was characterized by X-ray photoelectron spectroscopy (XPS).

## Methods

**Fig. 1:**
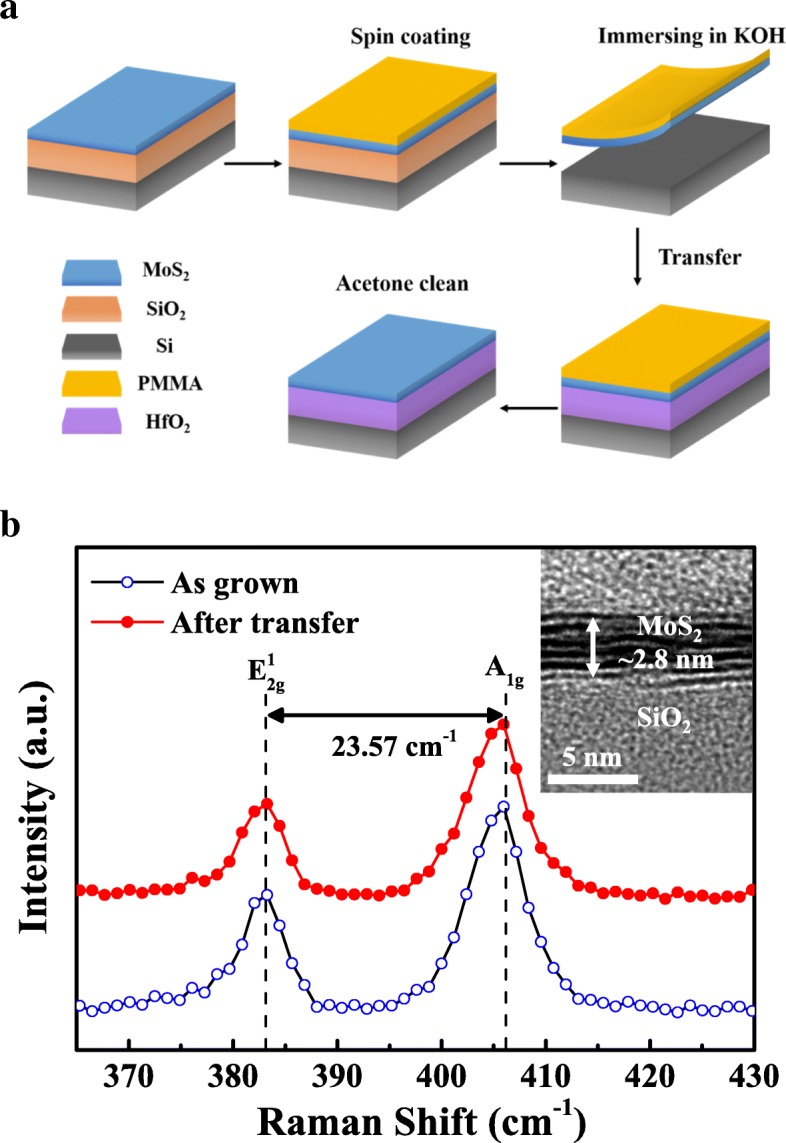
**a** Process flow of PMMA-assisted wet transfer method for the MoS_2_/ALD-HfO_2_ heterojunction formation. **b** Respective Raman spectra of as-grown and transferred MoS_2_ film. The inset is the cross-section transmission electron microscopy images of as-grown MoS_2_ on SiO_2_/Si substrate

The SiO_2_ (280 nm)/Si wafer was alternately cleaned with acetone and isopropanol by ultrasonic cleaning for each 10 min, followed by deionized water rinse and N_2_ dry. The few-layer MoS_2_ films were deposited on SiO_2_/Si substrate by chemical vapor deposition (CVD) using precursors of MoO_3_ (0.08 mg, 99%, Alfa Aesar) and S powder (1 g, 99%) [20, 21]. After the growth procedure, the MoS_2_ film would be transferred to HfO_2_/Si substrate by the poly (methyl methacrylate) (PMMA) method [22], as depicted in Fig. 1a. In this process, PMMA was first spin-coated on MoS_2_/SiO_2_/Si samples as a supporting layer. Then, the samples were immersed in KOH solution for etching away the SiO_2_, after which the MoS_2_ layer with PMMA would float to the top of the solution. In the end, the PMMA layer would be dissolved in acetone after the sample was transferred onto HfO_2_/Si substrate. The HfO_2_ films were grown on the silicon wafer by atomic layer deposition (ALD) at a temperature of 200 °C using Hf [N (CH_3_)(C_2_H_5_)]_4_ [tetrakis (ethylmethylamido) hafnium, TEMAH] and H_2_O vapor as precursors [23, 24]. During the optimization process of the plasma treatment time, it was found that the nitrogen would diffuse into the oxide greatly after 70 s nitridation treatment by SIMS measurements, which would severely deteriorate the oxide quality. While the plasma treatment time is 30 s, no obvious N peak at the oxide surface was observed from the SIMS results. For the control sample, 50 s N_2_ plasma treatment was implemented on HfO_2_/Si substrate at a pressure of 3 Pa before the MoS_2_ transfer. Under the plasma condition, the resultant N dose is about 8.4 × 10^14^ atoms/cm^2^ estimated from the secondary ion mass spectrometry (SIMS) results. And the concentration of nitrogen was calculated to be about 1.5% after nitridation based on the XPS data. Four samples 1–4# were prepared for XPS measurements: 1# few-layer MoS_2_ film on SiO_2_/Si substrate (few-layer MoS_2_), 2# thick HfO_2_ film on Si substrate (bulk HfO_2_), 3# transferred MoS_2_ film on as-grown HfO_2_/Si substrate (as-grown MoS_2_/HfO_2_ heterojunction), and 4# transferred MoS_2_ film on N_2_ plasma-treated HfO_2_/Si substrate (nitrided MoS_2_/HfO_2_ heterojunction).

## Results and Discussions

**Fig. 2:**
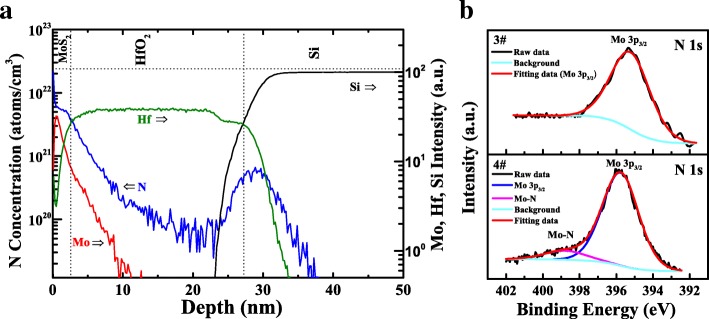
**a** SIMS depth profiles of transferred MoS_2_ film on nitrided HfO_2_/Si substrate. **b** N 1s XPS spectra for MoS_2_/HfO_2_ heterojunctions with and without nitridation treatment, respectively

RENISHAW inVia Raman spectroscopy was employed to characterize the Raman spectra of few-layer MoS_2_ film before and after transfer procedure, as illustrated in Fig. 1b. Two Raman peaks can be seen at around 382.86 cm^−1^ and 406.43 cm^−1^, corresponding to the in-plane (${E}_{2g}^1$) and out-of-plane (*A*_1*g*_) modes, respectively [25, 26]. It was found that there is nearly no Raman shift in ${E}_{2g}^1$ and *A*_1*g*_ mode frequencies after transfer process, indicating minimal structure modification. The frequency difference (*∆k*) between ${E}_{2g}^1$ and *A*_1*g*_ mode was deduced to be about 23.57 cm^−1^, designating around four to five layers of MoS_2_ film [27]. As shown in the inset of Fig. 1b, the thickness of MoS_2_ film was verified to be approximately 2.8 nm by high-resolution transmission electron microscope (HRTEM), which is in consistent with the abovementioned Raman spectra. Moreover, we presented SIMS depth profiles of transferred MoS_2_ film on nitrided HfO_2_/Si substrate. SIMS measurement was performed on a Physical Electronics ADEPT 1010 SIMS instrument with Cs primary ion beam at the energy of 1 keV, in which positive ions were collected and charge compensation was carried out. In this SIMS measurement, the nitrogen element was quantified while the other elements (Mo, Hf, and Si) are only meant as layer markers and not quantified. As illustrated in Fig. 2a, the depth profiles for transferred MoS_2_ film on nitrided HfO_2_/Si substrate were determined by SIMS, in which signals of main components represented by Mo, N, Hf, and Si are plotted against the depth. The spreading of N into the HfO_2_ layer was observed, which could be intrigued by the N injection into the underlying layer during primary beam bombards or plasma treatments. It is also worth noting that depth profiles near the surface layer are normally complicated and meaningless because of the surface contamination and surface effects, e.g., the abnormal intensity of N element near the surface [28]. The higher signal of N profile near the HfO_2_/Si interface could be ascribed to that the nitrogen tends to diffuse to the HfO_2_/Si interface, leading to the accumulation of N near the interface [29]. The tail of Mo in HfO_2_ film could be mainly caused by primary beam bombardments in SIMS measurements [30]. Figure 2b illustrates the respective N 1s XPS spectra for sample 3# and 4#; the high-intensity peaks for both heterojunctions were Mo 3p_3/2_ while a low-intensity peak at ~ 395.80 eV was detected for the nitrided heterojunction, indicating the formation of Mo-N bonding [31].

**Fig. 3:**
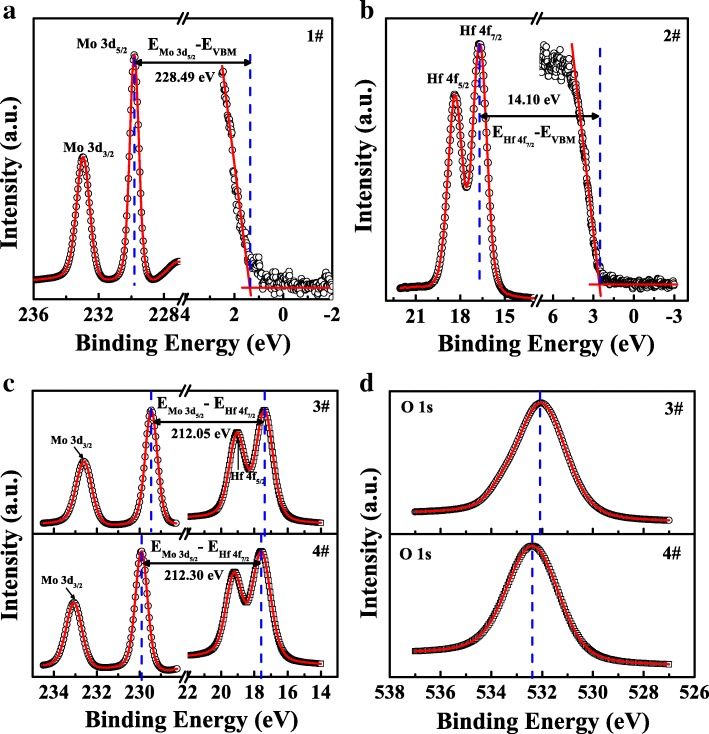
**a** XPS spectra of Mo 3d CL and valence band for the few-layer MoS_2_. **b** XPS spectra of Hf 4f CL and valence band for bulk HfO_2_. XPS spectra of **c** Mo 3d, Hf 4f, and **d** O 1s CLs for transferred MoS_2_ film on bulk HfO_2_ with/without nitridation treatment

To obtain the band alignments between few-layer MoS_2_ and HfO_2_ with and without nitridation treatment, XPS measurements with a step of 0.05 eV were carried out on VG ESCALAB 220i-XL system using a monochromatic Al Kα X-ray source (hν = 1486.6 eV). The constant pass energy was set at 20 eV. Additionally, the standard C 1s (284.8 eV) was used for binding energy (BE) calibration [32]. To evaluate VBO values for MoS_2_/HfO_2_ heterojunctions, Mo 3d and Hf 4f core levels (CLs) were selected for sample 1–4#, respectively. Figure 3a presents the XPS narrow scan of Mo 3d and valence band spectra from sample 1# [33]. Thus, the binding energy difference (BED) between Mo 3d_5/2_ core level and valence band maximum (VBM) for sample 1# was calculated to be 228.49 ± 0.1 eV. Figure 3b illustrates the CLs of Hf 4f_7/2_ and VBM for sample 2#; the corresponding BED was determined to be 14.10 ± 0.1 eV. Figure 3c depicts the measured XPS spectra of Mo 3d and Hf 4f CLs for MoS_2_/HfO_2_ heterojunctions with/without nitridation treatment. It is noted that the Mo 3d_5/2_ CL shifted from 229.45 ± 0.05 eV for sample 3# to 229.90 ± 0.05 eV for sample 4#. This could be ascribed to that a nitridation interfacial layer was introduced at the MoS_2_/HfO_2_ interface after plasma treatment, resulting in the abovementioned Mo-N bonding. With the presence of Mo-N bonding, the consequent charge transfer between Mo and N elements contributed to the measured Mo 3d_5/2_ CL shift. Additionally, the Hf 4f_7/2_ CL of 17.40 ± 0.05 eV for sample 3# was shifted to a higher binding energy of 17.60 ± 0.05 eV for sample 4# while O 1s also showed a shift of 0.20 eV to a higher BED, as shown in Fig. 3d. These peak shifts implied the downward band bending at the HfO_2_ surface, which could be interpreted as that the nitrogen plasma induced donor-like defects for HfO_2_ [34]. Based on the Kraut method [35], the VBO (*∆E*_*V*_) values can be calculated from the following equation:

1 $$ \Delta  {E}_V=\left({E}_{\mathrm{Mo}\ 3{\mathrm{d}}_{5/2}}^{\mathrm{Mo}{\mathrm{S}}_2}-{E}_{\mathrm{VBM}}^{\mathrm{Mo}{\mathrm{S}}_2}\right)-\left({E}_{\mathrm{Hf}\ 4{\mathrm{f}}_{7/2}}^{{\mathrm{Hf}\mathrm{O}}_2}-{E}_{\mathrm{VBM}}^{{\mathrm{Hf}\mathrm{O}}_2}\right)-{\Delta  E}_{\mathrm{CL}} $$

where ${E}_{\mathrm{Mo}\ 3{\mathrm{d}}_{5/2}}^{\mathrm{Mo}{\mathrm{S}}_2}$ and ${E}_{\mathrm{VBM}}^{\mathrm{Mo}{\mathrm{S}}_2}$ are binding energies of Mo 3d_5/2_ CL and VBM for MoS_2_, ${E}_{\mathrm{Hf}\ 4{\mathrm{f}}_{7/2}}^{{\mathrm{Hf}\mathrm{O}}_2}$ and ${E}_{\mathrm{VBM}}^{{\mathrm{HfO}}_2}$ are binding energies of Hf 4f_7/2_ CL and VBM for ALD-HfO_2_, *∆E*_CL_ =${E}_{\mathrm{Mo}\ 3{\mathrm{d}}_{5/2}}^{\mathrm{Mo}{\mathrm{S}}_2}-{E}_{\mathrm{Hf}\ 4{\mathrm{f}}_{7/2}}^{{\mathrm{Hf}\mathrm{O}}_2}$ refers to the BED between Mo 3d_5/2_ and Hf 4f_7/2_ CLs for ALD-HfO_2_/MoS_2_ heterojunctions. Hence, the *∆E*_*V*_ of MoS_2_ on ALD-HfO_2_ with and without nitridation treatment were calculated to be 2.09 ± 0.1 and 2.34 ± 0.1 eV, respectively.

**Fig. 4:**
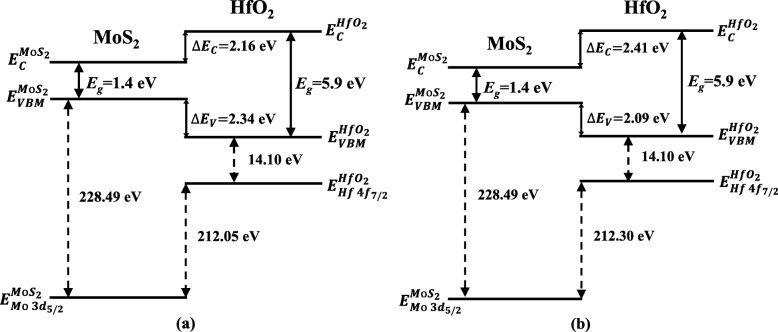
Band diagrams of MoS_2_/HfO_2_ heterojunction **a** without nitridation treatment and **b** with nitridation treatment

To assess the influence of N_2_ plasma treatment on the conduction band offset (CBO, *∆E*_*C*_) between ALD-HfO_2_ and few-layer MoS_2_, the bandgaps of 5.9 ± 0.1 eV for HfO_2_ and 1.4 ± 0.1 eV for MoS_2_ were used here, respectively [7, 36]. Thus, the CBO can be attained by the following equation:

2 $$ {\Delta  E}_C={E}_g^{{\mathrm{HfO}}_2}-{E}_g^{\mathrm{Mo}{\mathrm{S}}_2}-{\Delta  E}_V $$

where ${E}_g^{{\mathrm{HfO}}_2}$ and ${E}_g^{\mathrm{Mo}{\mathrm{S}}_2}$ are the bandgaps of HfO_2_ and MoS_2_, respectively. According to Eq. (2), the *∆E*_*C*_ between MoS_2_ and ALD-HfO_2_ with and without nitridation treatment were calculated to be 2.41 ± 0.1 and 2.16 ± 0.1 eV, respectively. The corresponding band diagrams are illustrated in Fig. 4. Remarkably, both VBO and CBO values of these two heterojunctions provide excellent electron and hole confinements, ensuring their suitability for MoS_2_-based FETs [37]. Moreover, the nitrided heterojunction has a higher CBO compared with unnitrided heterojunction, which is better for n-channel FETs applications.

## Conclusions

In conclusion, the XPS measurements revealed that the band alignment at the MoS_2_/HfO_2_ interface could be modified by introducing nitridation to HfO_2_ surface prior to stacking MoS_2_ film. The CBO and VBO were determined to be 2.16 ± 0.1 and 2.34 ± 0.1 eV for the unnitrided MoS_2_/HfO_2_ heterojunction, whereas the CBO was altered up to 2.41 ± 0.1 eV and the VBO was altered down to 2.09 ± 0.1 eV for the nitrided MoS_2_/HfO_2_ heterojunction, respectively. A nitridation interfacial layer was introduced at the interface, which was found to result in the Mo-N bonding formation. Additionally, the nitrogen plasma could induce donor-like defects, leading to the surface band bending for HfO_2_. In this way, the interfacial band alignment engineering would supply promising routes toward the flexible deign and optimization of modern electronics.

## Data Availability

The datasets supporting the conclusions of this manuscript are included within the manuscript.

## Abbreviations

ALD: Atomic layer deposition
BE: Binding energy
BED: Binding energy difference
CBO: Conduction band offset
CL: Core level
CVD: Chemical vapor deposition
FET: Field-effect transistor
HfO2: Hafnium oxide
HRTEM: High-resolution transmission electron microscope
MoS_2_: Molybdenum disulfide
PMMA: Poly (methyl methacrylate)
SIMS: Secondary ion mass spectrometry
SL: Single-layer
TEMAH: Tetrakis (ethylmethylamido) hafnium
TMDC: Transition metal dichalcogenide
VBM: Valence band maximum
VBO: Valence band offset
XPS: X-ray photoelectron spectroscopy
